# Nosocomial Drug-Resistant Bacteremia in 2 Cohorts with Cryptococcal Meningitis, Africa

**DOI:** 10.3201/eid2004.131277

**Published:** 2014-04

**Authors:** Radha Rajasingham, Darlisha Williams, David B. Meya, Graeme Meintjes, David R. Boulware, James Scriven

**Affiliations:** University of Minnesota, Minneapolis, Minnesota, USA (R. Rajasingham, D. Williams, D.B. Meya, D.R. Boulware);; Makerere University, Kampala, Uganda (D. Williams, D.B. Meya);; University of Cape Town, Cape Town, South Africa (G. Meintjes, J. Scriven);; Imperial College London, London, UK (G. Meintjes);; Liverpool School of Tropical Medicine, Liverpool, UK (J. Scriven)

**Keywords:** bacteremia, bacteria, nosocomial, sepsis, drug resistance, bloodstream infection, fungi, cryptococcal meningitis, Uganda, South Africa

**To the Editor:** Cryptococcal meningitis is the second leading cause of AIDS-related deaths in Africa. The prolonged hospitalization necessary for optimal management may predispose severely immunocompromised persons to hospital-acquired infections. Limited data are available for sub-Saharan Africa regarding multidrug-resistant infections (*1**,**2*). We hypothesized that bacteremia was a major cause of death.

We reviewed bacteremia episodes in cryptococcal meningitis cohorts in Kampala, Uganda (n = 115 episodes) and Cape Town, South Africa (n = 72) during November 2010–April 2013. Data were obtained from the prospective cryptococcal optimal antiretroviral therapy timing trial (www.clinicaltrials.gov:NCT01075152), a randomized strategy trial assessing optimal antiretroviral therapy timing (n = 142) and another prospective observational cohort in Cape Town (n = 45).

We enrolled HIV-infected adults who had a first episode of cryptococcal meningitis diagnosed by cerebrospinal fluid culture or cryptococcal antigen testing. Standardized treatment was in accordance with World Health Organization (WHO) guidelines: amphotericin deoxycholate, 0.7–1.0 mg/kg/d for 14 days, and fluconazole, 800 mg/d, requiring a minimum 14-day hospitalization (*3*). Each person provided written informed consent. Institutional review board approval was obtained.

Blood cultures were obtained in accordance with physician discretion, typically with new onset fever (>38°C) unrelated to amphotericin. Two aerobic blood cultures were obtained from 1 peripheral site and not from central catheters. BACTEC (Becton Dickinson, Franklin Lakes, NJ, USA) or BacT/ALERT (BioMérieux, Durham, NC, USA) bottles were incubated at 37°C in automated instruments for 5 days. Drugs were given empirically at physician discretion and adjusted after culture/susceptibility results were obtained.

Each bacteremic episode was classified as a true pathogen, contaminant, or indeterminate on the basis of clinical scenario and bacterial isolates. Data extraction from case report forms elicited demographics, microbiology results, antimicrobial drug therapy, and clinical outcomes. We determined risk factors between cases and controls who had cryptococcal meningitis without bacteremia/sepsis.

Descriptive statistical analysis reported median and interquartile range (IQR). Risk was expressed as odds ratio with 95% CIs calculated by logistic regression. Significance was defined as p<0.05 by Fischer exact test (SPSS 21; IBM, Armonk, NY, USA). Variables with p<0.10 by univariate analysis were entered in a multivariable model.

We evaluated 187 persons with cryptococcal meningitis who had a median CD4 count of 27 cells/μL (IQR 9–76). Forty-three blood cultures were prepared for 40 patients with febrile episode(s), of which 37 were positive. Median time from admission to suspected bacteremia was 14 days (IQR 9–17 days). All episodes were detected >72 h after admission and classified as nosocomial bacteremia. Seven isolates were considered contaminants because clinical improvement occurred without appropriate therapy. Thus, 30 cultures for 28 persons (cohort incidence 15%) were classified as true bacteremia with compatible clinical syndrome. Twenty-three bacteremic episodes occurred in Kampala (incidence 18%). Seven episodes occurred in 5 patients in Cape Town (incidence 7%).

The most frequent microbiologic etiologies were *Klebsiella pneumoniae* (9 episodes), *Staphylococcus aureus* (8), and *Pseudomonas* spp. (3) (Table). Methicillin-resistant *S. aureus* constituted 6 of 8 *S. aureus* isolates.

**Table:** Characteristics of 28 patients with cryptococcal meningitis, Uganda and South Africa, November 2010–April 2013*

Patient	Blood culture isolate	Hospital	30-d outcome	Ceftriaxone susceptibility†	Fluoroquinolone susceptibility†
1	*Staphylococcus aureus* (MRSA)	K	Died	R	NT
2	*S. aureus* (MRSA)	K	Survived	R	NT
3	*S.. aureus* (MRSA)	C	Survived	R	S
4	*S. aureus* (MRSA)	C	Survived	R	R
5	*S. aureus* (MRSA)	C	Died	R	S
6	*S. aureus* (MSSA)	K	Survived	S	NT
7	*S. aureus* (MSSA)	K	Survived	S	NT
8‡	*S. aureus* (MRSA)	C	Survived	R	R
8	*Enterobacter cloacae*	C	Survived	R	S
9	*Klebsiella pneumoniae*	K	Survived	R	R
10	*K. pneumoniae*	K	Died	R	R
11	*K. pneumoniae*	K	Survived	S	S
12	*K. pneumoniae*	K	Died	R	S
13	*K. pneumoniae*	K	Died	R	R
14	*K. pneumoniae*	K	Died	R	R
15	*K. pneumoniae*	K	Survived	R	R
16	*K. pneumoniae*	C	Survived	R	NT
17	*K. pneumoniae*	C	Died	R	S
18	*Pseudomonas putida*	K	Died	S	S
19	*Pseudomonas aeruginosa*	K	Survived	R	R
20	*Pseudomonas* spp.	K	Died	R	NT
21	*Salmonella* spp.	K	Survived	S	S
22	*Salmonella* spp.	K	Died	S	S
23	*Burkholderia cepacia*	K	Survived	R	NT
24	*B. cepacia*	K	Survived	S	S
25	*Citrobacter freundii*	K	Died	R	S
26	*Acinetobacter baumanii*	K	Survived	R	S
27	*Enterobacter* spp.	K	Died	R	R
28‡	*Enterobacter cloacae*	C	Survived	R	S
28	*Stenotrophomonas maltophilia*	C	Survived	R	R

*Sixteen (57%) of 28 patients survived; 7 (23%) of 30 isolates were susceptible to ceftriaxone, and 13 (54%) of 24 isolates were susceptible to fluoroquinolone. MSRA, methicillin-resistant *S aureus*; K, Kampala, Uganda; R, resistant; NT, not tested; C, Cape Town, South Africa; S, sensitive; MSSA, methicillin-sensitive *S. aureus*. †Drug sensitivity testing performed by using the Kirby-Bauer method in Cape Town, South Africa, and a Phoenix Automated Microbiology System (Becton, Dickinson, Franklin Lakes, NJ, USA) in Kampala, Uganda. ‡Two patients each had 2 episodes of bacteremia.

Ceftriaxone was the most common empiric drug used, for which 23 (77%) of 30 isolates were resistant. Eleven (46%) of 24 isolates were resistant to ciprofloxacin. Among bacteremic patients, 12 (43%) of 28 died within 30 days after hospitalization. The 30-day mortality rate for persons with cryptococcal meningitis but without bacteremia was 30% (47/158); 1 patient was lost to follow-up. Thus, the estimated attributable mortality rate for bacteremia was 13% (odds ratio 1.8, 95% CI 0.78–4.0, p = 0.17) compared with patients without bacteremia during their initial hospitalization.

Case–control comparisons identified no risk factors for bacteremia (Technical Appendix). Although 21 (70%) of 30 bacteremia episodes were preceded by phlebitis at a peripheral intravenous site, phlebitis caused by amphotericin was also common in patients without bacteremia (49%), but these percentages did not differ statistically.

Accurate data regarding incidence of nosocomial infections in Africa are lacking. A systematic review by WHO in 2011 that assessed published data for 1995–2009 identified only 2 high-quality studies. WHO estimated a prevalence of 2.5%–14.8% for nosocomial infections and a cumulative incidence of up to 45.8% in some areas (*4*) and recommended surveillance to estimate the rates of nosocomial infection. WHO acknowledges that health care–associated infections are causes of prolonged hospitalizations, increased antimicrobial drug resistance, financial burdens on health care systems, and causes of excess illness and death (*5*).

Limitations of our study include the retrospective design and inability to identify predictive risk factors for bacteremia. Given the differences in bacteremia incidence between our 2 sites, findings are probably not generalizable to all clinical settings in Africa. However, these findings identify a clinical problem.

The incidence of nosocomial bacteremia was 15% in our hospitalized cryptococcal meningitis cohort at a median time of 14 days after hospitalization. The most frequent etiologies were *S. aureus* and *K. pneumonia*. Less than 25% of isolates were sensitive to ceftriaxone, a standard empiric drug used throughout Africa. Further prospective studies are needed to determine the prevalence and risk factors for nosocomial infections and prevalence of multidrug resistance among hospitalized persons in resource-limited areas.

Technical Appendix Demographic data for persons with cryptococcal meningitis by nosocomial bacteremia status, and risk factors for bacteremia in a combined cryptococcal cohort, Cape Town, South Africa, and Kampla, Uganda, November 2010–April 2013.

## References

[1] Allegranzi B, Nejad SB, Combescure C, Graafmans W, Attar H, Donaldson L, Burden of endemic health-care–associated infection in developing countries: systematic review and meta-analysis. Lancet. 2011;377:228–41. 10.1016/S0140-6736(10)61458-4 21146207

[2] Paterson DL, Ko W-C, Gottberg Von A, Mohapatra S, Casellas JM, Goossens H, International prospective study of *Klebsiella pneumoniae* bacteremia: implications of extended-spectrum beta-lactamase production in nosocomial infections. Ann Intern Med. 2004;140:26–32 . 10.7326/0003-4819-140-1-200401060-00008 14706969

[3] World Health Organization. Rapid advice: diagnosis, prevention and management of cryptococcal disease in HIV-infected adults, adolescents, 2011 [cited 2014 Jan 7]. www.who.int/hiv/pub/cryptococcal_disease2011/en/26110194

[4] Bagheri Nejad S, Allegranzi BB, Syed SBS, Ellis BB, Pittet DD. Health-care-associated infection in Africa: a systematic review. Bull World Health Organ. 2011;89:757–65. 10.2471/BLT.11.088179 22084514 PMC3209981

[5] World Health Organization. Report on the burden of endemic health care–associated infection worldwide, 2011 [cited 2014 Jan 7]. http://whqlibdoc.who.int/publications/2011/9789241501507_eng.pdf

