# Supplementary material for: Nosocomial Drug-Resistant Bacteremia in 2 Cohorts with Cryptococcal Meningitis, Africa
Source: Emerg Infect Dis. 2014 Apr;20(4):722–4. doi: 10.3201/eid2004.131277 (PMC3966372; doi:10.3201/eid2004.131277)
Supplement: Technical Appendix — Demographic data for persons with cryptococcal meningitis by nosocomial bacteremia status, and risk factors for bacteremia in a combined cryptococcal cohort, Cape Town, South Africa, and Kampla, Uganda, November 2010–April 2013. [file 13-1277-Techapp-s1.pdf]

Article DOI: http://dx.doi.org/10.3201/eid2004.131277

Nosocomial Drug-Resistant Bacteremia in 2 Cohorts with Cryptococcal Meningitis, Africa

Technical Appendix

Technical Appendix Table 1. Demographic data for persons with cryptococcal meningitis by nosocomial bacteremia status, Kampala, Uganda and Cape Town, South Africa*

Study site, baseline demographics	Without bacteremia	With bacteremia	Univariate p value
Kampala, Uganda			
No.	94	21	NA
Age, y	35 (30–40)	36 (31–43)	0.37
Male sex	43 (46)	15 (71)	0.052
CD4 count, cells/μL	16 (7–70)	17 (6–60)	0.71
HIV viral load, log_{10} copies/mL	5.4 (5.2–5.7)	5.6 (5.5–5.8)	0.024
Leukocytes x 10^9/L	3.6 (2.6–4.9)	4.2 (3.1–6.0)	0.27
Thrombophlebitis	14 (47)†	13 (62)	0.39
Deaths at 10 wk	34 (36)	11 (52)	0.22
Cape Town, South Africa			
No.	65	7	NA
Age, y	36 (28–43)	34 (23–42)	0.66
Male sex	35 (54)	5 (71)	0.45
CD4 count, cells/μL	61 (16–98)	14 (11–43)	0.028
HIV viral load, log_{10} copies/mL	5.1 (4.6–5.7)	5.4 (4.8–5.5)	0.80
Leukocytes x 10^9/L	5.0 (3.4–6.2)	3.5 (2.2–5.0)	0.11
Thrombophlebitis	20 (50)†	6 (85)	0.11
Deaths at 10 wk	22 (35)‡	2 (29)	0.99

*Values are median (interquartile range) or no. (%) unless otherwise indicated. NA, not applicable. p values were calculated for continuous variables by using the Mann-Whitney U and for categorical variables by using the Fisher exact test.
†Detailed chart extraction was performed for thrombophlebitis on 30 persons without bacteremia in Kampala (n = 30) and on 40 persons in in Cape Town matched within 1 mo of diagnosis of a case-patient with bacteremia.
‡Two persons were lost to follow-up.

Technical Appendix Table 2. Risk factors for bacteremia in a combined cohort with cryptococcal infections, Cape Town, South Africa, and Kampala, Uganda*

Baseline demographics	Without bacteremia, n = 159	With bacteremia, n = 28	Univariate p value	Adjusted odds ratio (95% CI)	Multivariate p value
Cape Town	65 (90)	7 (9.7)	NA	Referent	NA
Kampala	94 (82)	21 (18)	0.14	1.4 (0.53–3.8)	0.49
Age, y	36 (30–42)	36 (30–42)	0.60	ND	NA
Male sex	78 (49)	20 (71)	0.039	2.4 (0.98–5.9)	0.055
Weight, kg	54 (48–58)	59 (50–65)	0.14	ND	NA
Leukocyte x 10^9/L	3.9 (2.9–5.8)	4.0 (2.7–5.5)	0.89	ND	NA
CD4 count, cells/μL	30 (10–79)	17 (6–47)	0.082	0.90 (0.80–1.02)†	0.10
HIV viral load, log_{10} copies/mL	5.4 (5.0–5.7)	5.6 (5.4–5.7)	0.046	2.1 (0.76–5.7)	0.15
C-reactive protein at day 7, mg/L	93 (54–143)	126 (67–180)	0.17	ND	NA

*Values are no. (%) of median (interquartile range). NA, not applicable; ND, not determined. Univariate p values were calculated for continuous variables by using the Mann-Whitney U and for categorical variables by using the Fisher exact test. Adjusted odds ratios and multivariate p values were calculated by using logistic regression that included site and variables with p<0.10 by univariate analysis.
†Odds ratio for CD4 is per each 10 cell/μL CD4 increase.
